# Bridging the gap: an empirical study on learning motivation, pathways, and outcomes in China’s 3 + 4 integrated vocational-bachelor education program

**DOI:** 10.3389/fpsyg.2025.1565323

**Published:** 2025-10-17

**Authors:** Jieqiong Chen, Li Lin, Xikun Gai, Yangyang Zhang

**Affiliations:** ^1^Academic Affairs Office, Zhejiang University of Science and Technology, Hangzhou, China; ^2^School of Economics and Management, Zhejiang University of Science and Technology, Hangzhou, China

**Keywords:** 3 + 4 integrated vocational-bachelor education, vocational education, educational psychology, learning conditions, transfer examination, career planning, teaching alignment

## Abstract

**Introduction:**

China’s ongoing vocational education reform has highlighted the “3 + 4” integrated vocational–bachelor model as a key pathway for cultivating technical and skilled talent. Using Zhejiang University of Science and Technology as a case, this study examines students’ learning motivation, self-efficacy, career planning, stage transitions, and transfer examinations from the perspective of educational psychology.

**Methods:**

A mixed-methods design was adopted, combining questionnaire surveys with factor, path, and neural network analyses to capture both linear and nonlinear relationships, supplemented by ANOVA to test differences across majors and grade levels.

**Results:**

The results reveal that the transfer examination mechanism is the starting point of students’ learning trajectories: lenient standards reduce motivation in the vocational stage and exacerbate misalignment with undergraduate learning. Career and learning planning emerge as the strongest predictors of positive learning outcomes, supporting career construction theory by demonstrating the role of clear vocational identity in sustaining engagement. Effective stage transitions also interact with academic motivation to influence learning states, consistent with self-efficacy theory. Differences across majors and grades are significant, while gender and place of origin exert weaker effects.

**Discussion:**

The study concludes that optimizing transfer examinations, strengthening curriculum integration, embedding career planning throughout training, and providing differentiated support are essential to improving the “3 + 4” model. Beyond Zhejiang, these findings offer insights for similar reforms in other Chinese provinces and for international systems such as Germany’s dual education model and Singapore’s polytechnic–university pathway. By linking empirical evidence with educational psychology and vocational education theory, the study contributes to both the theoretical understanding and practical improvement of integrated vocational–bachelor education globally.

## 1 Introduction

In recent years, China’s vocational education reform has continued to deepen. The “3 + 4” integrated vocational–bachelor model, which links secondary vocational schools with higher vocational (associate and bachelor) or regular undergraduate institutions, has become a major focus for both society and government. This reform aims to break down institutional barriers between vocational and general education. It also seeks to build a seamless training pathway for cultivating technical and skilled talents. At the secondary vocational stage, students gain systematic professional knowledge and practical experience. These foundations help them adapt more effectively to the theoretical and comprehensive demands of undergraduate education (Allen, 2024; Blair and Raver, 2016; Nwakanma, 2024). Policy documents have reinforced this direction. The 2021 China Vocational Education Conference emphasized the need to integrate vocational and general education, build a vertically connected system, and promote horizontal alignment (Newint-Gori et al., 2023; Yu et al., 2025). In July 2024, the 20th Third Plenary Session of the Communist Party of China called for “accelerating the construction of a vocational education system that integrates vocational and general education with industry–education collaboration.” Later that month, the Ministry of Education convened a meeting on modern vocational education reform, reaffirming the goal of “deepening industry–education collaboration and promoting vocational–general integration.” Together, these initiatives highlight the strategic importance of the integrated training model. It serves as a critical pathway to improve the quality of vocational education, increase its attractiveness, and provide clear development routes for multi-level technical and skilled talents.

Within the framework of national policies, Zhejiang Province has taken proactive steps in recent years. It has continuously advanced the integration of secondary vocational education with higher vocational and undergraduate education. Leveraging its economic and industrial structure, the province has pursued the “triple chain integration” of training chains, innovation chains, and support chains. At the same time, it has refined a modern vocational education system that is vertically integrated, horizontally aligned, and gradient-linked. Since 2018, Zhejiang has piloted the “3 + 4” integrated long-term training program in specialized fields that require long training cycles and advanced skills (Liu, 2024). The program is built on collaboration between secondary vocational schools and application-oriented universities. This partnership ensures close alignment in training objectives, curricula, and assessment systems (Wang and Ma, 2024). Over time, it has established an integrated system of curricula, textbooks, practical training, and evaluations. The goal is to achieve “3 + 4 > 7” in talent development, meaning that 7 years of integrated training yields results greater than the sum of its parts. According to the latest statistics, the program has expanded rapidly. By 2024, it had grown from 8 undergraduate institutions, 15 secondary vocational schools, and 590 enrollment slots to 26 undergraduate institutions, 77 secondary vocational schools, and 3,185 enrollment slots. This expansion has significantly increased its scale and influence, drawing widespread attention.

However, challenges persist in the implementation of the “3 + 4” integrated program. Common issues include insufficient student motivation (Edwards et al., 2024), lack of initiative (Sanchez et al., 2023), poor alignment between secondary (Halabieh et al., 2022) and undergraduate curricula, and weaknesses in the design and assessment processes of the transfer examination (Grote et al., 2021). These problems manifest not only in students’ academic performance but also in their level of engagement, self-confidence, and long-term career planning (Mydin et al., 2021). Surveys across several institutions reveal that in undergraduate programs with higher requirements for foundational knowledge (such as electrical engineering, mechanical design, and automation), the difficulty of undergraduate courses increases sharply (Amalu et al., 2023; Li, 2022). Students with weak foundational knowledge often develop resistance or anxiety toward courses such as advanced mathematics, university physics, and college English, leading to high failure rates and passive learning attitudes (Clark, 2023; Pundak et al., 2009).

From the perspective of educational psychology, learning motivation, academic self-efficacy, and learning burnout are key factors affecting students’ academic performance and professional identity (Abbasi et al., 2021; Kim et al., 2018). According to Self-Determination Theory, intrinsic motivation is driven by the fulfillment of basic needs for autonomy, competence, and relatedness (Dunn and Zimmer, 2020; Kondja et al., 2024). Schools and teachers must provide supportive environments to help students gain control over learning tasks, thereby fostering positive engagement (Han, 2021; Sökmen, 2021). For “3 + 4” students, practical skills gained in secondary vocational education, if not acknowledged and extended in undergraduate education, can lead to reduced competence and interest. Similarly, Bandura’s theory of self-efficacy emphasizes that students’ perception of their ability to complete academic tasks directly impacts their effort and persistence (Poluektova et al., 2023; Resnick, 2008; Schunk and DiBenedetto, 2016). If students perceive the increased breadth and depth of knowledge in undergraduate education as overwhelming or receive inadequate support, their self-efficacy declines, exacerbating motivational deficits (Natale, 2024).

Existing research on the “3 + 4” integrated program primarily focuses on educational policy, school-enterprise cooperation mechanisms, curriculum design, and talent development outcomes (Wei et al., 2022; Yang, 2018). While many studies recognize the program’s positive impact on extending the training cycle, strengthening school collaboration, and enhancing the appeal of vocational education (Hanushek et al., 2017), they also highlight issues of misalignment in teacher resources, curriculum design, practical training conditions, and students’ psychological needs between the two stages. Nevertheless, gaps remain in three areas: first, empirical studies on students’ learning conditions are limited, especially from the perspective of educational psychology, exploring the effects of motivation, self-efficacy, and learning burnout; second, most existing studies rely on qualitative methods or descriptive statistics, lacking causal path analysis or predictive modeling to systematically examine the interaction between students’ characteristics, attitudes, and performance; third, insufficient attention has been paid to the differentiated learning behaviors and psychological needs of “3 + 4” students across various majors, grades, and cognitive levels, weakening the applicability of current findings. This study, using Zhejiang University of Science and Technology as a case, conducts an in-depth empirical investigation with the following objectives:

First, to explore students’ attitudes, motivations, self-efficacy, and career awareness during the undergraduate stage through questionnaires and interviews, identifying the root causes of insufficient learning initiative;

Second, to clarify the logical relationships among six key dimensions—stage transitions, transfer examinations, teacher support, learning resources, professional cognition, and career planning—by employing factor analysis and path analysis, and to construct a causal path model influencing students’ academic performance;

Third, to utilize neural network analysis to examine the complex interactions between students’ characteristics and psychological factors based on variables such as major, grade level, and academic foundation, providing a scientific basis for differentiated teaching and refined management.

The innovative contributions of this study are threefold: first, it integrates educational psychology into the study of the “3 + 4” program, combining quantitative and qualitative methods to reveal psychological challenges faced by students and propose targeted strategies for enhancing learning motivation and self-efficacy; second, it advances the methodological framework by employing factor analysis, path analysis, and neural network analysis to connect students’ academic performance with psychological variables, transitioning from descriptive statistics to causal relationships and predictive modeling; third, grounded in the practical experiences of Zhejiang University of Science and Technology, it offers macro-level policy suggestions for optimizing transfer examinations and curriculum alignment while addressing diverse needs across majors and grade levels with individualized teaching and psychological support. These findings not only provide actionable insights for improving the “3 + 4” integrated program in China but also offer valuable references for global vocational education reform and transformation.

## 2 Research design and method

### 2.1 Research design

This study focuses on the learning conditions and educational psychological characteristics of students in the “3 + 4” integrated vocational-bachelor education program, using first- and second-year students from five integrated majors at Zhejiang University of Science and Technology as the primary research subjects. The aim is to comprehensively investigate the current status and influencing factors of learning motivation, career planning, stage transitions, and learning planning among integrated program students at the undergraduate level using a mixed-method approach. The specific research design is as follows:

First, in terms of sample selection, this study targets first- and second-year students because these stages represent a critical transition period from secondary vocational education to the undergraduate education system (Felder and Brent, 2005; Schechter, 2018). During this time, students’ learning attitudes and psychological characteristics are relatively pronounced, while the difficulty of courses, academic requirements, and school management practices undergo significant changes after the transition. Therefore, students from five integrated majors, including Electrical Engineering and Automation, Digital Media Technology, and Mechanical Design and Manufacturing Automation, were selected, covering a total of 403 participants to ensure the representativeness and validity of the sample.

Second, regarding research methods, this study primarily uses questionnaires supplemented with focus groups and interviews, forming a comprehensive research model that combines quantitative and qualitative approaches (Radović-Marković, 2023). The questionnaire consists of 33 items, employing a combination of a five-point Likert scale and open-ended, non-quantitative questions. The items focus on key elements such as learning motivation, career planning, learning planning, learning development, and learning outcomes after the transition. The five-point Likert scale effectively quantifies variables such as students’ learning attitudes, motivation levels, and career identity, while open-ended questions allow researchers to capture richer individual experiences and subjective perceptions. The questionnaires were distributed to students by class advisors and counselors to gather insights into the primary challenges students face in adapting to undergraduate learning, as well as their opinions and suggestions regarding teaching and management practices.

Third, in terms of data collection and processing, the study employed a combination of online and offline methods to distribute and collect questionnaires, aiming to ensure both the quantity and quality of responses. After data collection, the data were cleaned and screened, including the removal of incomplete or anomalous responses, followed by reliability and validity testing (Arndt et al., 2022). Descriptive statistical analysis was conducted using SPSS to provide a preliminary understanding of students’ overall learning conditions and psychological characteristics. Subsequently, to achieve the research objectives, factor analysis was used to extract dimensions and validate the structure of questionnaire items, identifying the core factors influencing students’ learning conditions and their internal relationships. On this basis, path analysis was employed to explore the causal relationships and influence paths among key factors, examining the direct and indirect effects of stage transitions, career planning, and learning planning on students’ learning motivation and learning outcomes. Finally, neural network analysis was introduced to explore the more complex and deeper non-linear interactions among multiple variables, constructing a predictive model of students’ learning performance. This provides quantitative evidence for targeted improvements in teaching management and student support services.

### 2.2 Survey sample

This study distributed 403 questionnaires, ultimately collecting 326 valid responses, resulting in an effective response rate of 80.89%, which indicates a relatively high sample recovery rate and reasonable representativeness. Regarding grade distribution, first-year students accounted for 66.26% of the sample, while second-year students made up 33.74% (Table 1). This reflects a relatively lower retention and engagement rate among second-year students in the “3 + 4” integrated program, which is also associated with the program’s focus on concentrated activities and assessments during the first year. Since first-year students are in the transition phase from secondary vocational education to undergraduate education, their learning experiences in the new environment and courses are typically more pronounced. Thus, data from this group are particularly valuable for understanding the initial learning conditions and transitional processes within the program.

**Table 1:** Sample distribution of “3 + 4” integrated program students’ learning conditions survey.

Variable	Category	Sample size	Percentage (%)
Grade	First-year	216	66.26
	Second-year	110	33.74
Gender	Male	252	77.3
	Female	74	22.7
Major	Mechanical design and manufacturing	59	18.1
	Automation	68	20.86
	Architectural electrical and intelligence systems	57	17.48
	Digital media technology	120	36.81
	Industrial design	22	6.75
Student origin	Rural	181	55.52
	Urban	145	44.48

In terms of gender distribution, male students significantly outnumber female students, accounting for 77.30 and 22.70%, respectively. This gender imbalance is common in engineering and technology-related majors and corresponds to the broader gender structure in vocational education enrollment in China. On the one hand, majors such as Mechanical Design and Manufacturing, Automation, and Architectural Electrical and Intelligence Systems typically have a higher proportion of male students in the industry. On the other hand, majors like Digital Media Technology and Industrial Design may attract more female students interested in artistic design and visual expression. However, the gender disparity remains significant across engineering-oriented majors.

Regarding major distribution, Digital Media Technology students represent the largest proportion (36.81%), followed by Automation (20.86%), Mechanical Design and Manufacturing (18.10%), Architectural Electrical and Intelligence Systems (17.48%), and Industrial Design (6.75%). Digital Media Technology’s higher proportion can be attributed to its relatively large enrollment scale in the integrated program. In contrast, Industrial Design has fewer students, partly due to its smaller enrollment plan and partly reflecting the preference of students and parents for engineering-focused majors in vocational-to-undergraduate pathways. The diversity of major distribution provides a good representation of the varying backgrounds of integrated program students, offering insights into differences in learning motivation, career planning, and academic progress.

In terms of student origin, rural students make up 55.52% of the sample, slightly exceeding urban students at 44.48%. This indicates that rural students remain a crucial source of enrollment in long-term vocational education programs. Due to disparities in urban and rural educational resources, family education investments, and growth environments (Liao et al., 2023), rural students may face more challenges in foundational learning, information access, and future career planning when transitioning to more demanding undergraduate courses. Schools need to provide more targeted support in course design, academic guidance, and career planning for rural students to address these challenges.

Additionally, the students in the sample come from seven secondary vocational schools in Zhejiang Province, each with a significant scale and distinctive features, including Hangzhou Zhongce Vocational School, Hangzhou Linping Senior Vocational School, Hangzhou Electronic Information Vocational School, Ningbo Vocational Education Center School, Anji Vocational Education Center, Quzhou Secondary Vocational School, and Zhejiang Information Engineering School. These schools are located in different regions of Zhejiang Province and demonstrate varied strengths in professional development, teaching conditions, and student growth. The diverse backgrounds of students from these schools bring a wide range of practical experiences and theoretical learning foundations from the secondary vocational stage. This diversity enables researchers to comprehensively understand the overall learning characteristics and needs of integrated program students, providing a rich comparative perspective for subsequent empirical analysis and policy recommendations.

### 2.3 Analytical methods

The quantitative analysis in this study is primarily based on data obtained from the questionnaire. In addition to basic reliability and validity testing methods, this study employs factor analysis (Factor Analysis), path analysis (Path Analysis), neural network analysis (Neural Network Analysis), and analysis of variance (ANOVA) to comprehensively explore the key factors influencing the learning performance of students in the “3 + 4” integrated program and their interactions, ensuring data quality throughout the process. The rationale for combining these methods lies in their complementary strengths. Factor analysis was first applied to reduce the dimensionality of the questionnaire items and extract latent constructs, thereby ensuring the scientific validity of the subsequent modeling. Based on the identified common factors, path analysis was conducted to examine the linear causal relationships among variables, allowing us to distinguish between direct and indirect effects and to validate theoretical assumptions regarding the impact pathways of transfer examinations, stage transitions, and career planning. Path analysis thus provides explanatory clarity and theoretical grounding for the model. Nevertheless, students’ learning conditions and psychological characteristics are not always governed by linear mechanisms alone. To capture these nonlinear and high-dimensional interactions, we further employed a Radial Basis Function (RBF) neural network. Unlike regression-based methods, the RBF model does not require strong assumptions about the form of relationships between variables. It is particularly suitable for uncovering complex, hidden patterns and for ranking the relative importance of predictors. In this study, the neural network provided predictive insights beyond the explanatory scope of path analysis, highlighting, for example, the disproportionate weight of career planning and transfer examinations in determining learning outcomes. Finally, analysis of variance (ANOVA) was used to test whether students’ learning performance differs significantly across categorical groups such as major, grade, gender, and place of origin. This step complements the earlier analyses by verifying whether background variables moderate the effects of psychological and institutional factors. In sum, each method plays a distinct role while complementing the others: factor analysis identifies valid constructs, path analysis clarifies linear causal mechanisms, neural networks reveal nonlinear dynamics and predictive importance, and ANOVA captures group heterogeneity. The combination of these approaches ensures that the study achieves both theoretical rigor (explanation of causal pathways) and practical robustness (prediction of complex outcomes and group-specific differences).

The specific steps and associated model formulas are outlined as follows:

#### 2.3.1 Factor analysis (factor analysis)

This study utilizes Principal Component Analysis (PCA) or Principal Axis Factoring (PAF) to extract common factors, complemented by Varimax orthogonal rotation or Promax oblique rotation. The aim is to “reduce dimensions” from the original 24 items of the questionnaire into fewer, more interpretable common factors. The basic model of factor analysis can be expressed as follows (Equation 1):


(1) $$X_{i}=\lambda_{i1}F_{i1}+\lambda_{i2}F_{i2}+\cdots +\lambda_{\mathrm{im}}F_{\mathrm{im}}+\varepsilon_{i}$$

Where X_i_ represents the i-th observed variable, F_1_, F_2_,…, F_m_ are the common factors, λ_ij_ are the factor loadings, and *ϵ*_i_ represents the unique factors or error terms. This process helps refine the dimensions into fewer, meaningful constructs that are critical for further analysis.

#### 2.3.2 Path analysis

To explore the interactions among the six dimensions identified and their impact on students’ learning outcomes, this study constructs and validates a path model. Path analysis can be considered an extension of multiple regression, with the basic form expressed as (Equation 2):


(2) $$Y=\beta_{1}X_{1}+\beta_{2}X_{2}+\cdots +\beta_{6}X_{6}+\varepsilon$$

Where Y represents the dependent variable (e.g., learning outcomes or academic performance), X_1_ to X_6_ represent the six factor scores, β are the regression coefficients, and ϵ represents the error term. By specifying structural relationships and creating path diagrams using SPSS or AMOS, it is possible to visually observe and test whether there are significant direct or indirect paths between the dimensions, as well as the magnitude and significance of path coefficients.

#### 2.3.3 Neural network analysis

After identifying linear relationships between factors, this study further investigates the complex nonlinear mechanisms affecting learning outcomes by using neural network analysis (Mansour et al., 2023). The six core dimensions are treated as input variables, while students’ certification outcomes and other performance metrics are treated as output variables, forming a BP (Backpropagation) neural network model. The input–output mapping of the BP neural network can be expressed as (Equations 3 and 4):


(3) $$\hat{y}=f(\sum_{j=1}^{m}w_{j}^{\left(1\right)}X_{j}+b^{\left(1\right)}$$

(4) $$\hat{Y}=g(\sum_{k=1}^{i}w_{k}^{\left(1\right)}{\hat{y}}_{k}+b^{\left(2\right)}$$

Where f and g are activation functions, $w_{j}^{\left(1\right)}$ and $\;w_{k}^{\left(1\right)}$ are connection weights, $b^{\left(1\right)}$ and $b^{\left(2\right)}$ are neuron biases. The backpropagation algorithm iteratively updates the weights to model the complex effects of multiple dimensions on learning outcomes. This provides a deeper analytical perspective for predicting students’ certification performance and subsequent academic progress.

#### 2.3.4 Analysis of variance (ANOVA)

Finally, this study uses ANOVA to examine the main effects and interaction effects of different background variables (e.g., major, grade level, gender, and place of origin) on the factor dimensions and learning outcome indicators. The basic ANOVA model is as follows (Equation 5):


(5) $$Y_{\mathrm{ijk}}=\mu +\alpha_{i}+\beta_{j}+{\left(\alpha \beta \right)}_{\mathrm{ij}}+\cdots +\varepsilon_{\mathrm{ijk}}$$

Where μ is the overall mean,$\alpha_{i}$represents the effect of the i-th factor level, $\beta_{j}$ represents the effect of the j-th factor level, ${\left(\alpha \beta \right)}_{\mathrm{ij}}$ represents the interaction effect of two factors, and$\varepsilon_{\mathrm{ijk}}\;$represents the random error term. By comparing the significance of differences in group means, this analysis identifies variations in aspects such as learning motivation and career planning across specific groups, providing insights for targeted educational interventions and policy development.

In summary, this study ensures the quality of questionnaire data through reliability and validity testing, uses factor analysis to extract key dimensions, and employs path analysis and neural network modeling to explore the linear and nonlinear effects on learning outcomes. Finally, through ANOVA, the study identifies differences across various student groups. Together, these methods provide multi-faceted empirical support for teaching practices and management decisions in the “3 + 4” integrated vocational-bachelor education program.

## 3 Empirical analysis results

### 3.1 Factor analysis

This study first conducted reliability and validity tests on the questionnaire data to ensure the reliability and applicability of the scale. In the reliability test, the Cronbach’s Alpha coefficient was 0.839, indicating good internal consistency of the questionnaire. In the validity test, the KMO value was 0.893, and Bartlett’s test of sphericity reached a significant level (*p* = 0.000), suggesting that the data was highly suitable for factor analysis and had good structural validity. Based on these results, factor extraction and subsequent empirical analysis were conducted.

The questionnaire data were analyzed using Principal Component Analysis (PCA) in SPSS, and Varimax rotation with Kaiser normalization was applied to ensure a clear and interpretable factor structure (Loewen and Gonulal, 2015). After eight iterations, the rotation converged, and six common factors were extracted. These six factors explained a total variance of 66.345%, indicating that the model effectively captures the key features of students’ learning conditions. The number of factors retained was determined using the criterion of eigenvalues greater than 1 combined with inspection of the scree plot, ensuring that only statistically meaningful constructs were preserved. Sampling adequacy was further confirmed by the high KMO value (0.893), and the significant Bartlett’s test indicated that the correlation matrix was not an identity matrix, thus meeting the assumption of factorability. These procedures strengthened the robustness of the extracted factors and provided assurance that the identified constructs are both reliable and theoretically interpretable. Based on the content and themes of the items within each factor, the factors were named as follows: Stage Transitions, Transfer Examinations, Career Planning and Development, Learning Motivation, Stage Learning Condition Changes, and Learning Planning. This analysis successfully constructed a theoretical model encompassing six dimensions, providing a solid data foundation for subsequent path analysis and neural network analysis. Table 2 presents the factor loadings after rotation, further verifying the rationality of the factor structure.

**Table 2:** Rotated component matrix.

Questionnaire items	Common factors					
	Stage transitions	Transfer examinations	Career planning and development	Learning motivation	Stage learning condition changes	Learning planning
1.1 Clear understanding of the training plan for each stage of the program	0.472					
1.2 Foundational courses in secondary vocational education support theoretical courses in undergraduate education	0.742					
1.3 Professional course textbooks at both stages are well-aligned and progressive	0.787					
1.4 Practical training at both stages has different focuses	0.762					
2.1 The difficulty of the transfer examination from secondary vocational to undergraduate education is low		0.524				
2.2 The transfer examination should have an appropriate elimination rate		0.839				
3.1 Clear career planning should be established during professional learning			0.689			
3.2 University instructors help students in secondary vocational education to establish career goals earlier			0.787			
3.3 Participation in competitions enhances comprehensive abilities			0.739			
3.4 The focus of training should differ between students in integrated programs and non-integrated programs of the same major			0.777			
3.5 Actively adapting to the university learning environment			0.524			
4.1 Academic pressure is high during the undergraduate stage				0.639		
4.2 Experiences of resistance or passivity arise when academic pressure is high				0.79		
4.3 Choosing the integrated program mainly to obtain an undergraduate degree				0.728		
4.4 The transfer examination is simple and does not require significant academic effort				0.756		
4.5 Actively studying a course is usually for the purpose of passing exams				0.735		
5.1 Learning motivation is relatively stronger during the undergraduate stage					0.505	
5.2 Relying on others to address academic or emotional issues					0.56	
5.3 Practical training during the undergraduate stage significantly enhances professional skills					0.65	
5.4 Development of professional knowledge and skills during the undergraduate stage					0.734	
5.5 Development of critical thinking, career identity, and sense of responsibility during the undergraduate stage					0.694	
6.1 Exploring professional training plans through various channels						0.446
6.2 Establishing a seamless pathway from secondary vocational to undergraduate and master’s education						0.706
6.3 Analyzing learning methods in-depth during the undergraduate stage						0.425

From the factor loading levels, 21 items have factor loadings greater than 0.5, while only 3 items have factor loadings below 0.5, but still exceed the critical threshold of 0.4. The six common factors identified after rotation align well with the initial design and assumptions of the questionnaire, further validating the scientific rigor of this empirical study.

From the results of the factor analysis, it was found that the transfer examination mechanism, the alignment of teaching between the secondary vocational and undergraduate stages, and early career planning are the three most significant common factors, collectively contributing 52.746% of the explained variance. These three factors are the primary determinants of the learning conditions of students in the “3 + 4” integrated program.

### 3.2 Path analysis

To further examine whether there are correlations and causal directions among the six common factors extracted through factor analysis, a path analysis was conducted.

**Figure 1:**
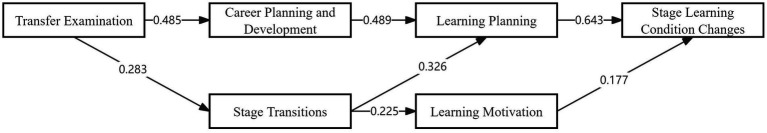
Weighted path diagram.

First, an in-depth review of recent project implementation details was conducted, and a model was developed based on the understanding and actual conditions of the project. A path direction diagram was drawn, and SPSS software was used to validate and calculate the model. The model was estimated using maximum likelihood estimation, and its overall fit was assessed through multiple indices, including χ^2^/df (<3.0), RMSEA (<0.08), CFI (>0.90), and TLI (>0.90), all of which met conventional thresholds for acceptable model fit. To ensure the robustness of regression-based estimation, multicollinearity among variables was examined using variance inflation factors (VIF), with all values below 5, indicating no serious multicollinearity. Normality of the observed variables was further checked through skewness and kurtosis statistics, which fell within the acceptable range (±2), supporting the assumption of approximate normal distribution. These procedures confirmed that the assumptions of path analysis were satisfied and that the estimated coefficients were statistically valid. After multiple iterations of modeling, validation, revisions, and recalculations, the weighted path diagram shown in Figure 1 was obtained.

The regression coefficients for each path in the model are shown in Table 3. From Table 3, it can be observed that the significance test results for all seven paths have *p*-values less than 0.01, indicating that these impact coefficients are reliable. The empirical results of the path analysis reveal that the transfer examination mechanism affects stage transitions and career planning and development, which in turn influence learning planning and ultimately lead to changes in students’ stage learning conditions. Similarly, stage transitions influence learning planning and learning motivation, which also ultimately point to changes in students’ stage learning conditions. All seven paths exhibit significant positive effects. Among these, the weight coefficients for four paths— the influence of the transfer examination mechanism on career planning and development, the influence of career planning and development on learning planning, the influence of learning planning on stage learning condition changes, and the influence of stage transitions on learning planning— all exceed 0.3. Notably, the coefficient for the influence of “learning planning on stage learning condition changes” reaches 0.643. Compared to the other three paths, these four sets of variable relationships demonstrate stronger causal effects.

**Table 3:** Model regression coefficients.

Path start node factor	→	Path termination node factor	Unstandardized coefficient	Standardized coefficient	Standard error	Significance level (*p*-value)
Transfer examination	→	Career planning and development	0.572	0.485	0.057	0.000***
Career planning and development	→	Learning planning	0.405	0.489	0.036	0.000***
Stage transitions	→	Learning planning	0.222	0.326	0.03	0.000***
Transfer examination	→	Stage transitions	0.407	0.283	0.076	0.000***
Stage transitions	→	Learning motivation	0.265	0.225	0.064	0.000***
Learning planning	→	Stage learning condition changes	0.872	0.643	0.055	0.000***
Learning motivation	→	Stage learning condition changes	0.138	0.177	0.032	0.000***

***Denotes significance at the 1% level. This table provides a detailed summary of the regression results for each path in the model, highlighting the significant direct and indirect effects between factors.

### 3.3 Radial basis function (RBF) neural network analysis

To observe the weight of the six common factor dimensions on learning outcomes, the six factors were used as independent variables, and certification results such as CET-4 (College English Test Band 4), which reflect students’ learning outcomes, were used as dependent variables. The relationships and impact levels between these variables were analyzed using an RBF neural network. The RBF neural network consisted of an input layer with six extracted factors, a hidden layer using Gaussian radial basis activation functions, and an output layer corresponding to students’ learning outcomes. The spread parameter was optimized through cross-validation, and weight updates were performed using a backpropagation algorithm. To guard against overfitting, the dataset was randomly split into training (70%), validation (15%), and testing (15%) subsets. Predictive accuracy was assessed by mean squared error (MSE) and correlation coefficients between predicted and observed values, confirming the stability of the model.

**Figure 2:**
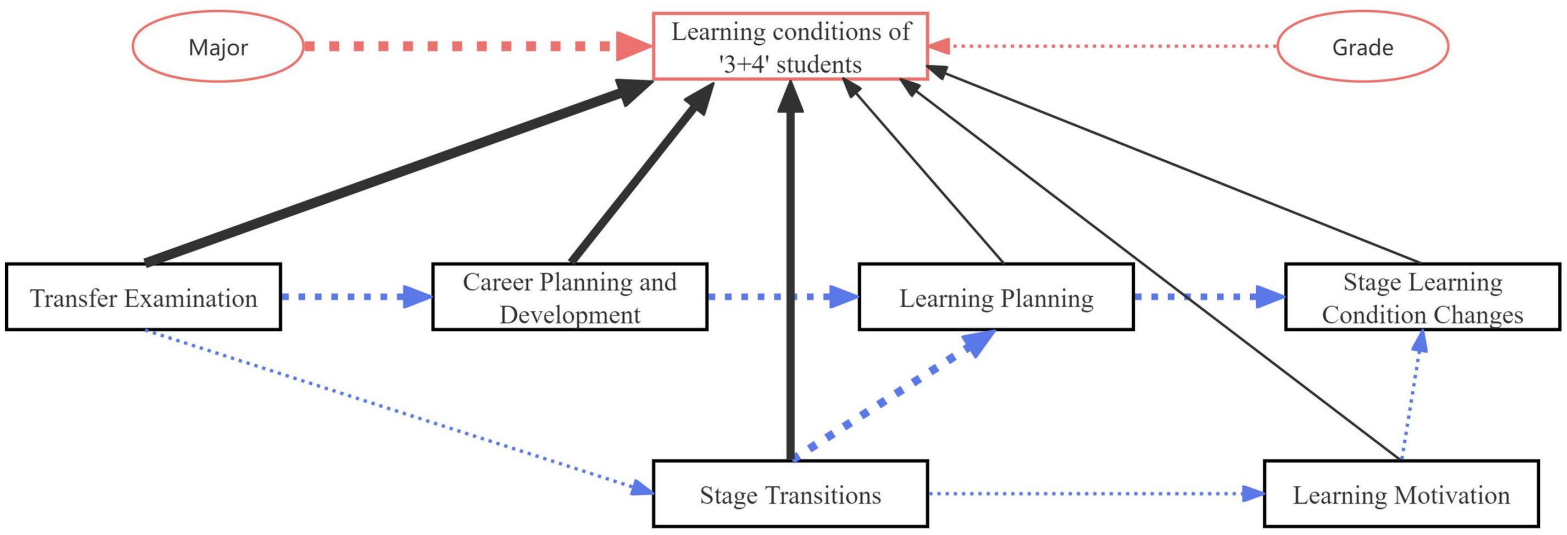
RBF neural network analysis diagram.

The RBF neural network employs a kernel function to map data from a low-dimensional space to a high-dimensional space, thereby resolving linear inseparability issues and yielding a linear relationship result. The simulated results are shown in Figure 2.

Through the calculations, we obtained normalized importance indicators to compare the importance of different independent variables (see Table 4). The normalized importance indicators standardize data with different dimensions, enabling a fair comparison between different indicators. This helps us better understand the influence of various independent variables on learning outcomes.

**Table 4:** Normalized importance of independent variables.

Independent variable	Importance	Normalized importance (%)
Transfer examination	0.212	78.40%
Learning planning	0.127	46.80%
Stage learning condition changes	0.204	75.30%
Learning motivation	0.082	30.20%
Stage transitions	0.104	38.40%
Career planning and development	0.271	100.00%

From the results of the neural network analysis, it is evident that the three independent variables—“Transfer Examination,” “Stage Learning Condition Changes,” and “Career Planning and Development”—hold higher levels of importance. This indicates that these variables have a significant impact on learning outcomes during the learning process. This may be because these factors are directly related to students’ learning motivation, goal-setting, and adjustments in learning strategies. Among these, “Career Planning and Development” ranks as the most important variable. If students place greater emphasis and expectations on their future career planning and development, they are more likely to adopt a positive attitude toward their own growth, resulting in better learning outcomes.

### 3.4 Analysis of variance (ANOVA)

To further examine whether the learning conditions of students in the “3 + 4” integrated program are related to different categorical variables, such as gender, grade, major, and place of origin, a multi-factor ANOVA was conducted using SPSS. For the ANOVA procedure, assumptions of normality and homogeneity of variance were explicitly examined. Shapiro–Wilk tests confirmed that the dependent variables approximated normal distributions, and Levene’s test was used to check the equality of variances. When the assumption of homogeneity was violated, Welch’s ANOVA was conducted as a robustness check. *Post hoc* pairwise comparisons were further performed using Tukey’s HSD test to identify specific group differences. The results are shown in Table 5. From the results of the F-test, it can be concluded that the significance levels (*p*-values) for the categorical variables “Grade” and “Major” are 0.021** and 0.001***, respectively, indicating statistical significance. These variables have a significant impact on learning outcomes, demonstrating main effects. On the other hand, the p-values for “Gender” (0.216) and “Place of Origin” (0.432) are not statistically significant, suggesting no main effects. Thus, “Major” and “Grade,” especially the “Major” factor, significantly influence students’ learning conditions, whereas “Gender” and “Place of Origin” are not directly associated with their learning conditions.

**Table 5:** Results of ANOVA.

Categorical variable	Sum of squares	Degrees of freedom	Mean square	*F*-value	*P*-value	*R* ^2^	Adjusted *R*^2^
Intercept	4.888	1	4.888	438.246	0.000***	0.067	0.046
Grade	0.06	1	0.06	5.418	0.021**		
Gender	0.017	1	0.017	1.535	0.216		
Major	0.203	4	0.051	4.546	0.001***		
Place of Origin	0.007	1	0.007	0.619	0.432		
Error	3.547	318	0.011	NaN	NaN		

***p < 0.01, **p < 0.05.

### 3.5 Comprehensive analysis and overall framework

**Figure 3:**
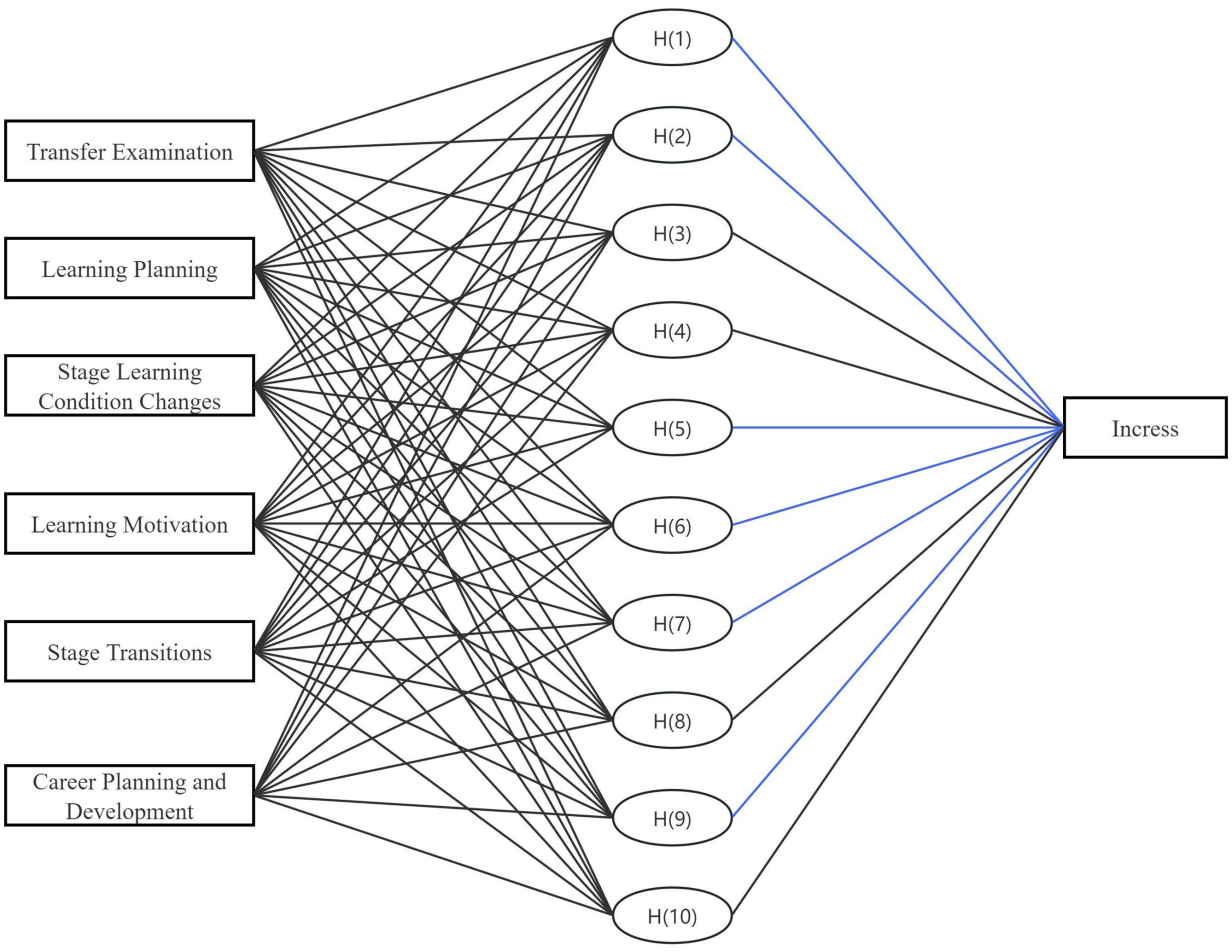
Overall mapping of the empirical analysis.

The comprehensive analysis framework is shown in Figure 3. From the figure, we observe that, in addition to the significant impacts of the “Major” and “Grade” factors on the learning conditions of “3 + 4” integrated program students, the six dimensions—Stage Transitions, Transfer Examinations, Career Planning and Development, Learning Motivation, Stage Learning Condition Changes, and Learning Planning—collectively determine the overall learning conditions of the students. Moreover, there are significant positive relationships among these six common factors.

The overall framework indicates that the learning conditions of “3 + 4” students are jointly influenced by the six common factor dimensions as well as the two additional factors, “Major” and “Grade.” The primary factor affecting the learning conditions of “3 + 4” students is the transfer examination mechanism. Over the past 3 years, the 100% transfer rate has impacted students’ career planning and development. Once students have career planning in place, they proceed to develop learning plans, which subsequently lead to adjustments in their learning conditions during the undergraduate stage. At the same time, the lack of an elimination mechanism has caused a disconnect between secondary vocational education and undergraduate education. Students in the secondary vocational stage lack sufficient motivation to learn. Upon transitioning to the undergraduate stage, the increased academic workload and lack of a strong theoretical foundation compel students to adjust their learning conditions during their undergraduate studies, thereby undermining the intended benefits of a long-term integrated education program.

Legend Explanation:

Black arrow represents the contribution of the six common factors, where thicker lines indicate a greater contribution to learning conditions.

Purple arrow represents the path correlations between the six common factors, where thicker lines indicate stronger causal relationships.

Red arrow represents the significance of multi-factor ANOVA results, where thicker lines indicate a greater impact on learning conditions.

## 4 Discussion

### 4.1 Optimize the transfer examination mechanism

The study confirms that the transfer examination mechanism is the most fundamental factor influencing students’ learning conditions, affecting not only their immediate motivation but also their long-term career planning and stage transitions. From the perspective of Self-Determination Theory, examinations with clear and challenging standards can enhance students’ sense of competence, thereby stimulating intrinsic motivation. However, Zhejiang’s current 100% pass rate for the transfer examination risks undermining this effect, as the absence of academic pressure diminishes students’ initiative during the vocational stage, a finding consistent with previous empirical work that shows excessively lenient assessments weaken self-regulation and persistence. By contrast, prior studies on vocational education reform suggest that moderately selective assessments, when linked to career advancement opportunities, create a balance between accessibility and rigor, encouraging sustained effort. To optimize the mechanism, two reforms are necessary: first, the subjects and evaluation criteria of the transfer examination should be closely aligned with the requirements of undergraduate curricula, ensuring that passing the exam reflects readiness for higher-level learning; second, an elimination mechanism should be introduced, such as setting minimum thresholds for individual subjects and raising the bar for combined academic and vocational assessments, to maintain a level of selectivity that sustains motivation. International comparisons provide valuable insights: Germany’s dual system links assessments to industry-recognized competencies, ensuring that progression is merit-based, while Singapore’s polytechnic–university pathway incorporates competitive selection that reinforces students’ motivation to achieve higher standards. These practices suggest that Zhejiang’s system could benefit from recalibrating its transfer examination policy to both preserve fairness and strengthen the quality of talent cultivation. Importantly, this mechanism illustrates how assessment design directly interacts with the motivational processes described in Self-Determination Theory: competence is enhanced when challenges are meaningful, but undermined when standards are absent. Thus, the transfer examination serves not only as a procedural requirement but also as a structural determinant of students’ motivational orientation, linking institutional policy to individual psychological outcomes.

### 4.2 Strengthen stage-based curriculum integration

The analysis reveals that poor alignment between the vocational and undergraduate stages is one of the most significant obstacles to sustaining student motivation and academic success, consistent with earlier studies emphasizing that discontinuities in curriculum design weaken learning confidence. From the perspective of vocational education alignment theories, coherent progression across stages ensures that students perceive their prior learning as meaningful and relevant, which in turn strengthens self-efficacy, as argued by Bandura’s framework. In Zhejiang, however, many students reported a sense of redundancy and disconnection between courses, leading to motivational decline, especially in science- and engineering-related majors that require a strong theoretical foundation. This pattern mirrors findings from international contexts, where gaps between vocational and higher education stages are associated with higher dropout rates. To address this issue, curriculum frameworks must be systematically redesigned to prioritize integration: vocational curricula should establish robust foundations that naturally lead into the theoretical demands of undergraduate study, while undergraduate courses should build progressively on vocational learning outcomes. A third-party evaluation mechanism could also be introduced to ensure that curricular coherence is objectively assessed and continuously improved. These recommendations are supported by international experiences: in Switzerland, applied universities implement vertically integrated curricula that emphasize progression from vocational competencies to higher-order theoretical skills, while in the United States, community college–university transfer systems use articulation agreements to guarantee seamless course transitions. Adapting such practices would not only improve Zhejiang’s “3 + 4” program but also enhance its international comparability and long-term sustainability. Theoretically, this reflects the role of continuity in sustaining self-efficacy: when learners can trace a clear trajectory from vocational learning to higher-level academic content, they perceive mastery experiences, which Bandura identifies as the strongest source of self-efficacy. Conversely, curricular disconnection undermines these mastery signals, weakening both confidence and motivation.

### 4.3 Establish comprehensive career planning

Career planning emerged as the strongest predictor of positive learning outcomes, validated across both path analysis and neural network modeling. This finding resonates strongly with career construction theory, which highlights the central role of vocational identity and future orientation in sustaining persistence and engagement. Students with clear career goals are better able to regulate their learning strategies, overcome motivational slumps, and align their efforts with long-term development pathways. Conversely, students without structured career guidance often display fragmented learning behaviors and diminished motivation over time, a problem observed in both Chinese and international contexts. Previous studies also indicate that the absence of career-oriented education weakens the perceived relevance of academic tasks, leading to disengagement. To address this, comprehensive career planning should be embedded throughout the “3 + 4” pathway: vocational-stage students should have access to orientation programs where university faculty participate in clarifying academic trajectories; undergraduate-stage students should engage in ongoing career seminars, industry-linked projects, and dual mentorship involving both academic advisors and enterprise mentors. These practices align with educational psychology, where long-term goal clarity enhances intrinsic motivation and reduces academic burnout. Internationally, Germany’s dual education system provides extended internships and structured career modules that tie academic knowledge directly to occupational identity, while Singapore’s polytechnic programs incorporate modular, industry-linked training that strengthens career readiness. By drawing on such models, Zhejiang’s program can provide continuous scaffolding for career development, ensuring that students maintain both academic engagement and professional motivation across the seven-year integrated pathway. In theoretical terms, the prominence of career planning underscores the explanatory power of career construction theory: vocational identity serves as a mediating variable linking institutional support (mentorship, internships) to psychological persistence. In this way, the observed empirical patterns are not incidental but reflect established psychological mechanisms of goal clarity, identity development, and future orientation.

### 4.4 Address the impact of majors and grades on learning conditions

The multi-factor analysis demonstrated that both major and grade significantly affect students’ learning conditions, with second-year students showing a marked decline in motivation. This finding corresponds to Bandura’s self-efficacy theory, which suggests that when learners face tasks exceeding their perceived competence, their confidence and persistence diminish. In engineering and technical majors, where theoretical demands rise sharply in the undergraduate stage, this effect is particularly pronounced, consistent with prior studies that link curricular difficulty to motivational decline. Meanwhile, first-year students often display higher enthusiasm due to the novelty of the transition, but without adequate scaffolding, their self-efficacy deteriorates in later years. To address these challenges, training plans must be revised according to disciplinary characteristics, ensuring that “3 + 4″ students are not treated identically to high school graduates. Professional training clusters tailored to the unique foundation of vocational students should be introduced, while academic supervision should be enhanced through collaboration between vocational and undergraduate faculty. Moreover, grade-specific interventions are necessary: first-year students require adaptation support and motivational reinforcement, while second-year students need targeted strategies to maintain confidence and resilience, such as peer mentoring, remedial courses, or psychological support services. International parallels underscore the importance of differentiation: South Korea’s Meister high schools implement grade-sensitive support to sustain motivation across transitions, and Germany’s applied universities provide specialized academic counseling for students in demanding majors. Extending these practices to Zhejiang and beyond would enhance the adaptability of the “3 + 4″ model and ensure its effectiveness across diverse institutional and cultural contexts. Linking this to theory, the variation across majors and grades illustrates how self-efficacy is a dynamic construct: it fluctuates in response to contextual demands and perceived mastery. The decline observed in second-year students exemplifies the vulnerability stage identified in developmental psychology, where rising task difficulty interacts with limited coping resources. Thus, major- and grade-specific differences are not only empirical observations but also theoretically predictable outcomes within self-efficacy and developmental frameworks.

## 5 Conclusion

This study conducted an empirical analysis of the learning conditions of students in the “3 + 4” integrated vocational–bachelor education program at Zhejiang University of Science and Technology, drawing on educational psychology theories and multiple analytical methods. Several key conclusions can be made.

First, the transfer examination mechanism functions as the starting point for shaping students’ learning behaviors and outcomes. While the current guaranteed transition policy lowers barriers to undergraduate study, it also weakens students’ intrinsic motivation and reduces their perceived urgency to learn, leading to misalignment between vocational and undergraduate stages. This finding is consistent with research in educational psychology showing that overly lenient assessments fail to activate the competence dimension of Self-Determination Theory, thereby limiting motivation. It also echoes earlier vocational education studies that emphasize the motivational benefits of moderately selective assessments. The result highlights the need to recalibrate transfer standards so that examinations encourage effort while ensuring fairness.

Second, career planning and learning planning play a central role in sustaining motivation and adaptability across stages. Students with clear career goals and structured learning plans demonstrated stronger initiative and greater resilience in meeting the demands of undergraduate study, which is consistent with career construction theory that emphasizes the importance of future orientation and vocational identity. Previous research has shown similar patterns in both Chinese and international contexts, but this study contributes by empirically demonstrating that career planning outweighs other predictors in integrated vocational–bachelor settings.

Third, effective stage transitions are essential for maintaining academic motivation and preventing passive adjustment. Redundant or poorly sequenced curricula undermine students’ ability to transfer strengths from the vocational stage into undergraduate learning, especially in majors requiring high levels of mathematics and science. This aligns with Bandura’s self-efficacy theory, which suggests that when learners perceive insufficient preparation, their confidence declines and burnout increases. Compared with earlier studies, this work adds evidence that motivational decline during transitions is not inevitable but can be mitigated by coherent curricular design and support systems.

Fourth, differences in major and grade have a significant impact on learning conditions, while gender and place of origin show weaker effects. Students in majors with more application-oriented curricula, such as Digital Media Technology, achieved better outcomes, reflecting the role of curricular design and difficulty in shaping learning motivation. Furthermore, while first-year students displayed enthusiasm during the initial transition, second-year students showed a marked decline in initiative, revealing the challenge of sustaining engagement in long-term integrated models. These findings are in line with international experiences—such as South Korea’s Meister high schools and Germany’s applied universities—where differentiated, stage-sensitive support is critical for sustaining self-efficacy over time.

In conclusion, this study not only confirms existing theories of motivation, self-efficacy, and career identity but also extends them into the context of integrated vocational–bachelor programs. While the model has improved the attractiveness of vocational education in China, it also exposes weaknesses in assessment design, curricular coherence, and sustained support mechanisms. Future reforms should prioritize optimizing the transfer examination mechanism, strengthening curriculum integration across stages, embedding career planning throughout the program, and providing differentiated support tailored to majors and grade levels. Beyond Zhejiang and China, the findings have broader implications for integrated education systems globally, offering lessons for countries with established pathways such as Germany, Singapore, and Switzerland. By linking empirical evidence with educational psychology and vocational education theory, this research contributes both to theoretical development and to the practical improvement of integrated vocational–bachelor education worldwide.

## Data Availability

The data presented in this study are available on request from the corresponding author. The data are not publicly available due to privacy. Requests to access the datasets should be directed to chenjq1980@126.com.
